# Degradation of Paracetamol by an UV/Chlorine Advanced Oxidation Process: Influencing Factors, Factorial Design, and Intermediates Identification

**DOI:** 10.3390/ijerph15122637

**Published:** 2018-11-25

**Authors:** Yen Hai Dao, Hai Nguyen Tran, Thien Thanh Tran-Lam, Trung Quoc Pham, Giang Truong Le

**Affiliations:** 1 Institute of Chemistry, Vietnam Academy of Science and Technology, 18 Hoang Quoc Viet, Cau Giay, Ha Noi 100000, Vietnam; dhy182@gmail.com (Y.H.D.); tltthien@hcmus.edu.vn (T.T.T.-L.); phamquoctrung0811@gmail.com (T.Q.P.); 2 Institute of Research and Development, Duy Tan University, Da Nang 550000, Vietnam

**Keywords:** paracetamol, UV/chlorine, reaction kinetics, response surface methodology, transformation products

## Abstract

The combination of a low-pressure mercury lamp and chlorine (UV/chlorine) was applied as an emerging advanced oxidation process (AOP), to examine paracetamol (PRC) degradation under different operational conditions. The results indicated that the UV/chlorine process exhibited a much faster PRC removal than the UV/H_2_O_2_ process or chlorination alone because of the great contribution of highly reactive species (^•^OH, ^•^Cl, and ClO^•^). The PRC degradation rate constant (*k*_obs_) was accurately determined by pseudo-first-order kinetics. The *k*_obs_ values were strongly affected by the operational conditions, such as chlorine dosage, solution pH, UV intensity, and coexisting natural organic matter. Response surface methodology was used for the optimization of four independent variables (NaOCl, UV, pH, and DOM). A mathematical model was established to predict and optimize the operational conditions for PRC removal in the UV/chlorine process. The main transformation products (twenty compound structures) were detected by liquid chromatography coupled to high-resolution mass spectrometry (LC-HRMS).

## 1. Introduction

Recently, pharmaceutical and personal care products (PPCPs) and polar pesticides—ubiquitously present in water bodies (i.e., groundwaters, rivers and lakes, hospital effluents, domestic effluents, pharmaceutical industries, and wastewater treatment plants)—have been identified and reported as emerging organic micropollutants. The elimination of residual PPCPs from the water environment (especially from municipal wastewater) is an urgent concern for the social and scientific communities. In essence, the majority of synthetic PPCPs constituents are difficult to effectively eliminate through biological degradation [1,2]. Paracetamol (PRC)—one of the most ubiquitous non-prescription drugs—is extensively used to relieve moderate intensity pain (i.e., headaches, muscle, and other minor pains) because of its antipyretic and analgesic properties [3]. Although PPCPs residues are typically detected in water bodies at trace levels ranging from a few nanograms per liter (ng/L) to several microgram per liter (μg/L) [4,5], their existence in an aquatic environment could deteriorate the water quality, cause ecotoxicity to living aquatic organisms, and negatively impact human health (particularly with long-term exposure) [2,6]. 

According to the previous literature, various potential approaches have been explored and applied to remove PPCPs from water media. They include photodegradation (i.e., using carbonaceous-TiO_2_ composites) [7], membrane bioreactors [8], adsorption (i.e., using carbon nanotubes) [9], aquatic plant-based systems (i.e., constructed wetlands) [10], ultrasonic treatment [11], biological treatment [2], ozonation [12], coagulation and sedimentation [13], electrochemical processes [14], and chlorination [15]. Among these methods, the combination of UV irradiation and chlorination is the most frequently used method in water and wastewater treatment for two purposes: disinfection (i.e., inactivating pathogenic microorganisms) and contaminant elimination (i.e., removing PPCPs). In addition, the chlorination disinfection coupled with UV irradiation have been widely used for the post-treatment of drinking water due to its excellent capacity in controlling taste, odor, and bacterial growth [16]. 

It has been reported that chlorine-based disinfectants (i.e., NaOCl) can produce some primary radicals (^•^OH and ^•^Cl) and chlorine-containing secondary radicals (Cl_2_^•−^, ClHO^•−^, and ClO^•^) that have been acknowledged as powerful oxidants [16,17]. Furthermore, reactive chlorine species (i.e., ^•^Cl, Cl_2_^•−^, and ClO^•^ as selective oxidants) can rapidly react with various compounds containing aromatic rings (i.e., benzene) [18] and electron-rich organic moieties (i.e., olefins, phenols, anilines, and deprotonated-amines) [19]. Therefore, the combination of UV irradiation and chlorination (UV/chlorine) turns into the advanced oxidation process (AOP). This combination can enhance the degradation efficiency of diffident kinds of organic pollutants, such as atrazine [20], ibuprofen [21], diuron [22], trimethoprim [23], phenacetin [15], benzoic acid [24], and clofibric acid [25,26]. It is also expected that such combination can remarkably enhance the degradation efficacy of paracetamol in water solutions.

Notably, depending on the target organic compounds, some transformative products, which are generated during the chlorination process, can be more toxic than the target parent compounds. As reported by many researchers, the ^•^OH and ^•^Cl radical groups played an important role in the decomposition pathways of organic compounds [15,20,22,23,25,26]. The existence of by-products generated from the degradation process of parent organic compounds through the UV/chlorine system has been thoroughly identified by the gas chromatography–mass spectrometry (GC-MS) or liquid chromatography–mass spectrometry (LC-MS). However, the combination of liquid chromatography-tandem mass spectrometry (LC-MS/MS) and Compound Discoverer 2.0 in identifying certain intermediate compounds generated during the degradation of organic compounds (especially paracetamol) has been limitedly reported in the scientific literature. 

Therefore, in this study, we aimed to: (1) investigate the effects of operational parameters on PRC degradation efficiency and rate in water media using the UV/chlorine process, (2) apply the response surface methodology to evaluate the interactions of four independent factors (i.e., UV, pH, NaOCl, and dissolved organic matter) on the PRC degradation process, and (3) postulate the probable degradation pathways of PRC under the UV/chlorine treatment by the LC-MS/MS methodology.

## 2. Materials and Methods 

### 2.1. Chemicals

Sodium hypochlorite solution (5% NaOCl), sodium chloride (NaCl), hydroxyl peroxide (H_2_O_2_), sodium sulphate (Na_2_SO_4_), sodium sulphite (Na_2_SO_3_), sodium hydroxyl (NaOH), bicarbonate (NaHCO_3_), and sulfuric acid (H_2_SO_4_) were purchased from Sigma-Aldrich (Sydney, Australia). Meanwhile, phosphoric acid, methanol, and acetonitrile of HPLC grade were obtained from Fisher Scientific (Waltham, MA, USA). All chemicals used in this work were of reagent grade, and they were directly used without further purification. Deionized water was used to prepare whole solutions in this study.

### 2.2. Experimental Procedures

**Figure 1:**
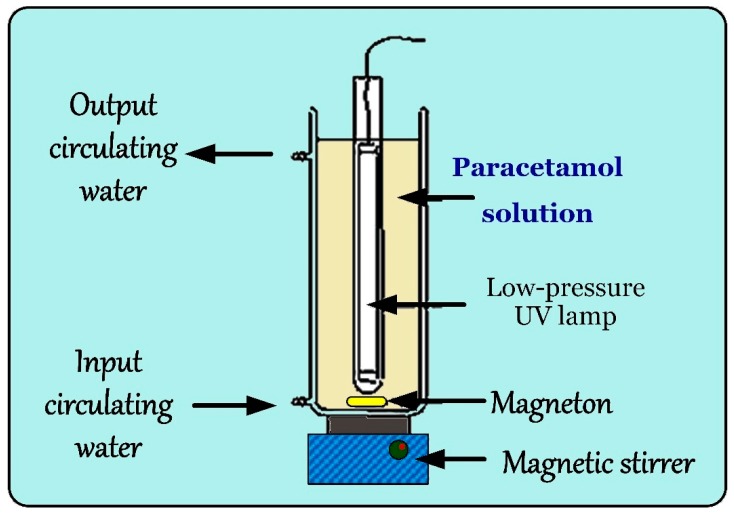
Experiential design for degradation of paracetamol by the UV/ chlorine system.

Experiments were carried out using a batch reactor system that is schematically drawn in Figure 1. Briefly, the reactor consists of a 2-L cylindrical double-wall jacketed vessel to circulate thermostatted water with an external circulating pump connected to a thermostatic water bath (25 ± 0.5 °C). The low-pressure mercury lamp (G6 T5, TUV 6W, 254 nm, Philips, Jena, Germany) was vertically fixed in the center of the reactor. The photon fluxes (*I*_o_) emitted from the UV lamp were measured by hydrogen peroxide actinometry using high concentrations of hydrogen peroxide (50–125 mM) to absorb all the photons received by the solution (49.7–102.2 mM) [27,28]. The calculated mean of *I*_o_ of Hg lamp was 3.41 × 10^−6^ (± standard deviation; SD = 0.105 × 10^−6^) Einstein/s. The reactor was covered by a black plastic film to protect the PRC aqueous solution from ambient light. The solution was homogeneously stirred by a magnetic stirrer at 400 rpm. The intensity of the UV lamp was adjusted by changing the number of UV lamps in the reactor. Solution pH value was adjusted using 0.5 M HClO_4_ or 0.5 M NaOH. The degradation of PRC by the UV/chlorine system was initiated by spiking a designed amount of NaClO into the initial PRC solution (~10 μmol/L). At target time intervals, approximately 1 mL of reaction solution was sampled for analyzing PRC concentrations and intermediates. The samples were quenched by adding sodium sulphite at twice the stoichiometric ratio in order to avoid further reactions.

### 2.3. Analytical Methods

Chlorine was determined by the standard *N,N*-diethyl-*p*-phenylenediamine (DPD) colorimetric method (4500-Cl G) [29]. The concentration of H_2_O_2_ was spectrophotometrically analyzed using the TiCl_4_ method [30]. Total organic carbon (TOC) was measured by a carbon analyzer (Multi N/C Analytik Jena, Jena, Germany). The concentration of PRC in solution was determined using HPLC (Dionex 3000 Thermo, Sunnyvale, California, USA) coupled with a photo-diode array. The column was a Hypersil Gold C-18 (150 mm × 2.1 mm × 3 µm, Thermo Fisher Scientific, Waltham, Massachusetts, USA). The intermediates were identified by a LC/HRMS Q-Exactive Focus system (Thermo). The MS/MS parameters were optimized as follows: sheath gas flow rate: 35, aux gas flow rate: 15, sweep gas flow rate: 1, spray voltage (kV): 3.4, capillary temperature: 320 °C, S-lens RF level: 50, aux gas heater temperature: 350 °C, and CE: 18. The used mobile phase includes solvent (A) H_2_O 0.1% Formic acid and solvent (B) CH_3_CN, with the gradient being 0 min: 5%B, 0–27 min: 5–95%B, 27–28 min: 95%B, 28–28.5 min: 5%B, and 28.5–30 min: 5%B. The signals were normally recorded in two modes, such as positive and negative in 30 min.

### 2.4. Data Analysis by Response Surface Methodology

The statistical software MODDE 12.1 trial (Umetrics, Malmö, Sweden) was used to create the experimental design, statistical analysis, and regression model. Response surface methodology (RSM) based on quadratic and cubic models with central composite circumscribed design (CCC) is composed of a full factorial design and star points (star distance: 2). The RSM was used to study the simultaneous effects of independent variables—UV photon fluxes (Einstein/s), pH, NaOCl (μM), and dissolved organic matter (DOM; mg/L)—on response functions for removal PRC efficiency from water solutions. The four independent variables (UV, pH, NaOCl, and DOM) were coded with *X*_1_, *X*_2_, *X*_3_, and *X*_4_, respectively; and each independent variable was divided into five levels (Table 1). The real values of the variable related to the coded variable are indicated in Equation (1). The relationship between the response functions and the coded variables is presented by a second-degree polynomial (Equation (2)). Furthermore, thirty-one combinations along with seven replicates of the central point were formed, corresponding to 24 experiments. The experiments with the coded and real values of the variables are shown in Table 1.

(1) $$\mathrm{Coded}\;\mathrm{variable}=\frac{X-X_{0}}{\lambda }$$

*Y* = *β*_0_ + *β*_i_∑*x*_i_ + *β*_ii_∑*x*^2^_i_ + *β*_ij_∑*x*_i_*x*_j_ (2)

where *X*_0_ is the real value of variables at the central level; *λ* is the step change of the variable; *Y* is a response function; *x*_i_ and *x*_j_ are independent variables; *β*_0_ is a constant; and *β*_i_, *β*_ii_, and *β*_ij_ are the linear, quadratic, and interactive coefficients, respectively.

### 2.5. Kinetic Degradation Modelling

The kinetic model for the UV/chlorine degradation of substances has been developed, and the kinetics can be described as the pseudo-first-order equation with respect to the contaminant concentration:

(3) $$\ln\frac{C_{o}}{C_{t}}=k_{\mathrm{obs}}t=(k_{\mathrm{chlorine}}+k_{\mathrm{radicals}}+k_{\mathrm{UV}})t$$

where *k*_obs_ is the observed rate constant of the pseudo-first order equation (1/s); *t* is the irradiation time (s); *C*_o_ and *C*_t_ are the initial and final concentrations of PRC contaminant (µM); and *k*_chlorine_, *k*_UV_, and *k*_radicals_ represent the degradation contributions of chlorine, UV, and reactive radicals, respectively.

## 3. Results and Discussion 

### 3.1. Comparison of PRC Degradation Efficiency by Different Operational Systems

**Figure 2:**
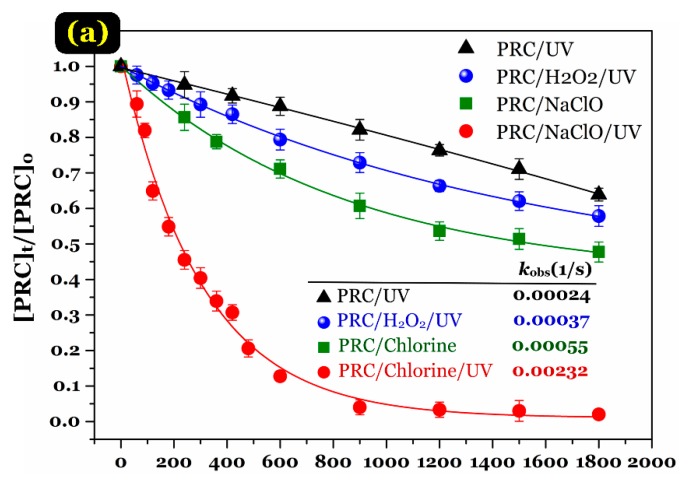

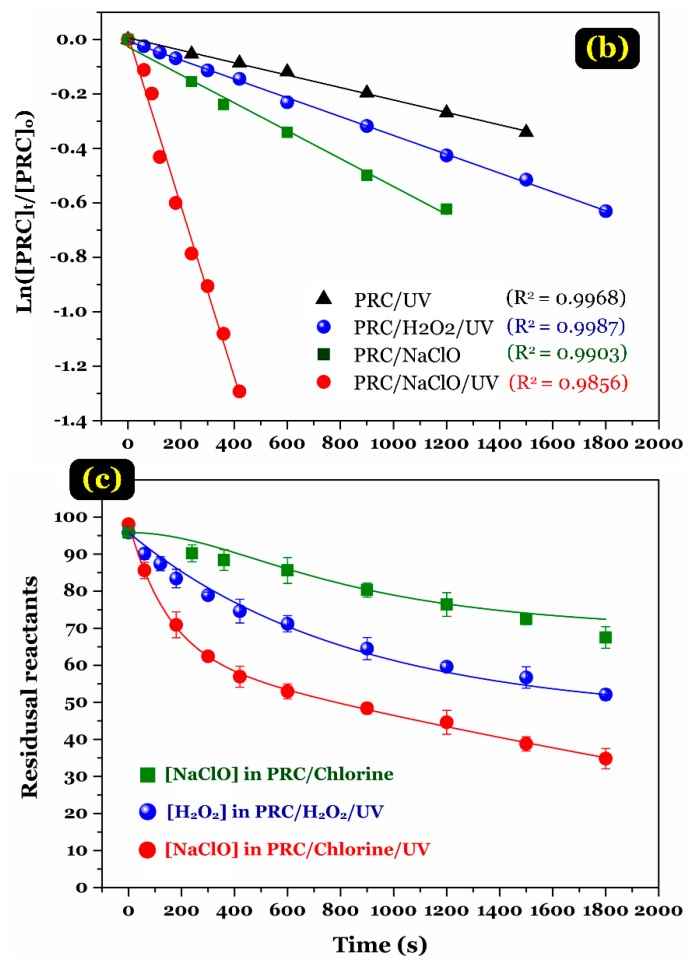
(**a**) Comparison of PRC degradation over time by the UV irradiation alone, UV/H_2_O_2_, chlorination alone, and UV/chlorine processes; (**b**) the plot of pseudo-first-order model (Experimental conditions: UV photon flux = 3.41 × 10^−6^ Einstein/s, [PRC]_o_ ≈ 10 µM, pH = 6.5, [NaClO] = 100 µM, and [H_2_O_2_] = 100 µM); and (**c**) time-dependent profile of the residual reactants.

The comparative time-dependent PRC degradation results achieved by the different processes (i.e., UV irradiation alone, chlorination alone, UV/H_2_O_2_ oxidation, and UV/chlorine oxidation) in pure water are shown in Figure 2a. The results demonstrated that the PRC degradation efficiency within 30 min by the different processes decreased the following order: UV/chlorine (~99%) > NaClO alone (~53%) > UV/H_2_O_2_ (~42%) > UV irradiation alone (~36%). Dark chlorination is usually used to degrade organic pollutants through the oxidation of free chlorine, which is similar to the finding of Zhu and co-workers [15] for phenacetin (~50%). As expected, the combination reaction of chlorination with UV irradiation (UV/chlorine) effectively removed PRC within 20 min (Figure 2a). In essence, when sodium hypochlorite is transferred into water solution, a hydrolysis process rapidly occurs to form hypochlorous acid (HOCl) and hypochlorite ion (OCl^−^) (Equations (4) and (5)). During the UV/chlorine process, HOCl and OCl^−^ are activated by the UV photolysis to simultaneously produce the main ^•^OH and ^•^Cl radicals (Equations (6)–(8)). The formed ^•^Cl radicals can react with the chloride ions (derived from the HOCl or OCl^−^ solution) to form Cl_2_^•−^ (Equation (9)) [24,26,31]. Those radicals can induce the PRC oxidation and sequentially generate some specific by-products. Notably, the previous study demonstrated that the contribution of Cl_2_^•−^ and O^•−^ radicals in the degradation of aromatic pollutants was negligible [24]:

NaOCl + H_2_O → Na^+^ + HOCl + OH^−^ (4)

HOCl ⇌ H^+^ + OCl^−^ (5)

HOCl + *hv* → ^•^OH + ^•^Cl (6)

OCl^−^ + *hv* → O^•−^ + ^•^Cl (7)

O^•−^ + H_2_O →^•^OH + OH^−^ (8)

^•^Cl + Cl^−^ ⇌ Cl_2_^•−^ (9)

Figure 2b gives information on the concentration of residual reactants after the elapsed time (~1/2 h) during the dark chlorination alone, UV/H_2_O_2_, and UV/chlorine processes. Clearly, the combined reaction of UV irradiation and chlorination consumed more chlorine (approximately twice) than chlorination alone. The result suggested that the majority of chlorine were used for the transformation of paracetamol under the UV/chlorine advanced oxidation process. Therefore, to determine observed rate constants (*k*_obs_) of the PRC degradation reactions, the oxidation reactants (NaClO and H_2_O_2_) were used with a higher amount to prevent any scavenging effects of active radicals by these reactants. As provided in Figure 2a, the PRC degradation rate constant (1/s) by the UV/chlorine process (0.00232/s) was approximately six-time faster than that by the UV/H_2_O_2_ one (0.00037/s). A similar finding was reported by Zhu and colleagues [15] for the degradation of phenacetin and Xiang and colleagues [21] for the degradation of ibuprofen. This is because the photodegradation quantum yield (*ϕ*; mol/Einstein) under the UV irradiation (*λ* = 254 nm) of HOCl (1–1.5 mol/E) and OCl^−^ (0.87–1.3 mol/E) was higher than that of H_2_O_2_ (0.5–1.0 mol/E) [26,31,32]. In other words, HOCl and OCl^−^ are is mild free radical scavengers compared to H_2_O_2_. 

### 3.2. Effect of UV Light Intensity on PRC Degradation

**Figure 3:**
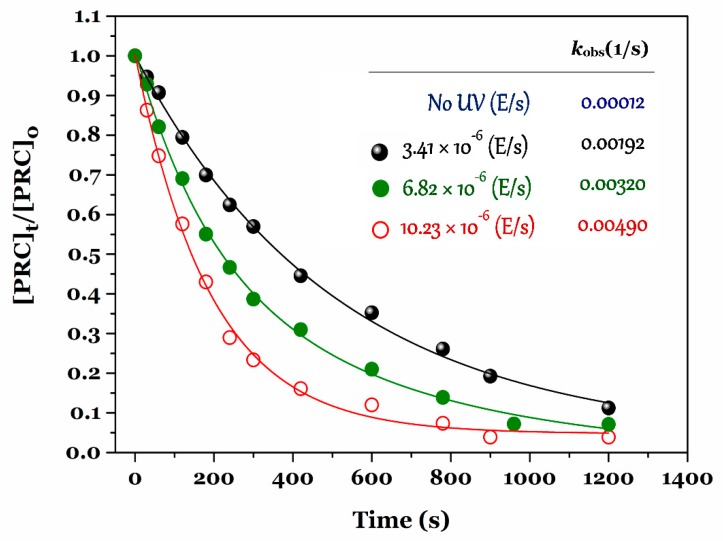
Effect of UV photon fluxes (Einstein/s) on the PRC degradation efficiency and rate constant by the UV/chlorine process (experimental conditions: [PRC]_o_ ≈ 10 µM, pH = 6.5, and [NaClO]_o_ = 100 µM).

Effects of UV intensity on the PRC degradation by the UV/chlorine process were conducted under different UV photon fluxes (×10^−6^ Einstein/s), such as 3.41, 6.82, and 10.23 (Figure 3). The degradation rate constant (*k*_obs_) increased the following order: 0.00192 < 0.00320 < 0.0049 (1/s) when the UV intensity increased from 3.41, to 6.82, and then to 10.23 (×10^−6^ Einstein/s), respectively. The result suggested that the PRC degradation rate constant was strongly dependent on the UV intensity; a higher UV intensity coincided with a faster PRC degradation in solution [21,33]. In this study, we selected the UV intensity of 3.41 × 10^−6^ (Einstein/s) for further experiments because of sufficient degradation and economic efficiency.

Notably, the degradation efficiency of PRC by the UV/chlorine advanced oxidation process can result from three contributions, including (1) the direct reaction of chlorine with PRC, (2) the photolysis of PRC under UV irradiation (254 nm), and (3) the reactive radicals generated from the photolysis process of chlorine. Table 2 presents the contribution percentages of UV, chlorine, and radicals on PRC degradation efficiency by the UV/chlorine process. The contributions of reactive radicals (^•^OH, ^•^Cl, and ClO^•^) were calculated as approximately 85%, proving that the predominant role of reactive radicals in the UV/chlorine decomposition of PRC. 

### 3.3. Effect of Chlorine Dosage on PRC Degradation

**Figure 4:**
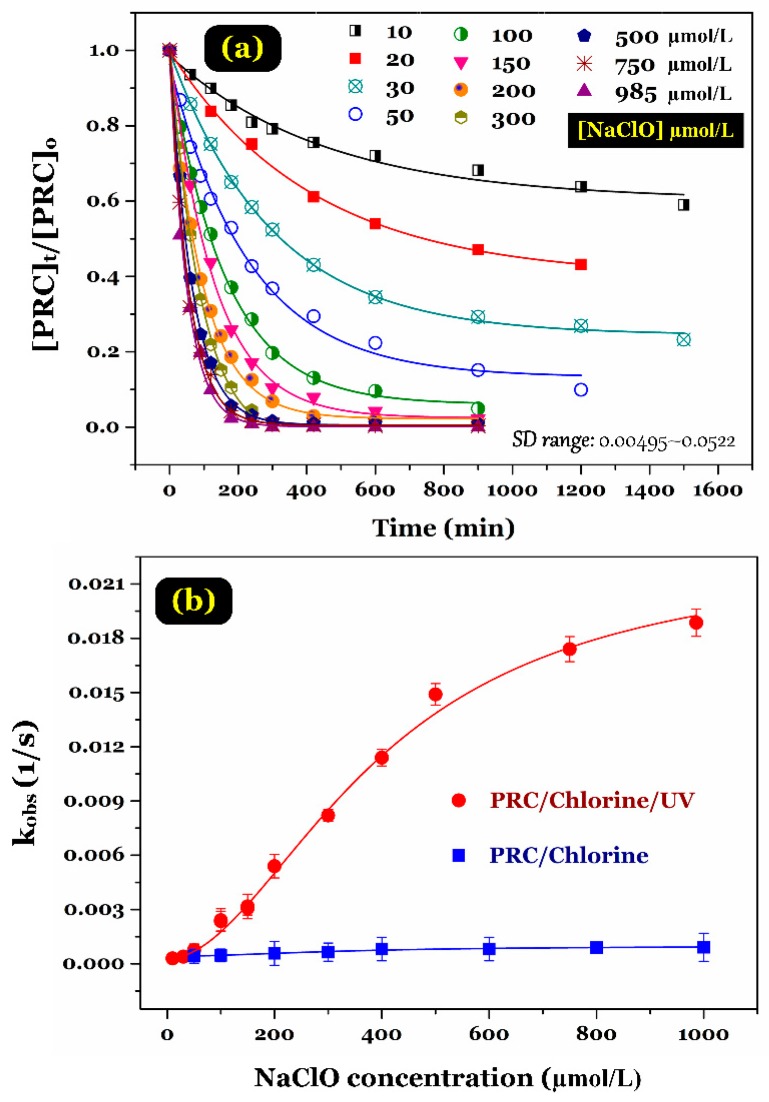
Effect of NaClO concentrations on (**a**) degradation efficiency and (**b**) degradation rate constant (Experimental conditions: UV photon flux = 3.41 × 10^−6^ Einstein/s, pH = 6.5, [NaClO]_o_ = 10–985 µM, and [PRC]_o_ ≈ 10 µM).

The time-dependent profiles of paracetamol degradation by the UV/chlorine process under different chlorine dosages ranging from 10 μmol/L to 985 μmol/L are displayed in Figure 4a. In general, an increase in chlorine dosage lead to increasing PRC degradation efficacy. The percentage of PRC degradation reached a constant value of approximately 98% when the chlorine dosage used was 100 μmol/L. The observed degradation rates of paracetamol under the UV and dark chlorination processes are illustrated in Figure 4b. Clearly, the *k*_obs_ values remarkably increased when the chlorine dosage increased, suggesting an increasing in the generation of ClO^•^ radicals. 

However, because of the variable chlorine dosage, the combined effects of two opposing aspects such as radical generation and scavenging could pose some complicated influence on the degradation rate of aromatic compounds. The upward trend of the reaction rate constant of PRC degradation by the UV/chlorine process is similar to the observations of some previous studies, such as trimethoprim degradation [23] and diuron degradation [22] under UV/chlorine process conditions. However, a dissimilar finding was reported by Fang and colleagues [24] for degradation of a micropollutant (benzoic acid) under UV/free chlorine process conditions. To avoid the residual concentrations, we selected a NaClO concentration of 100 μmol/L for the subsequent experiments.

### 3.4. Effect of Solution pH on PRC Degradation

**Figure 5:**
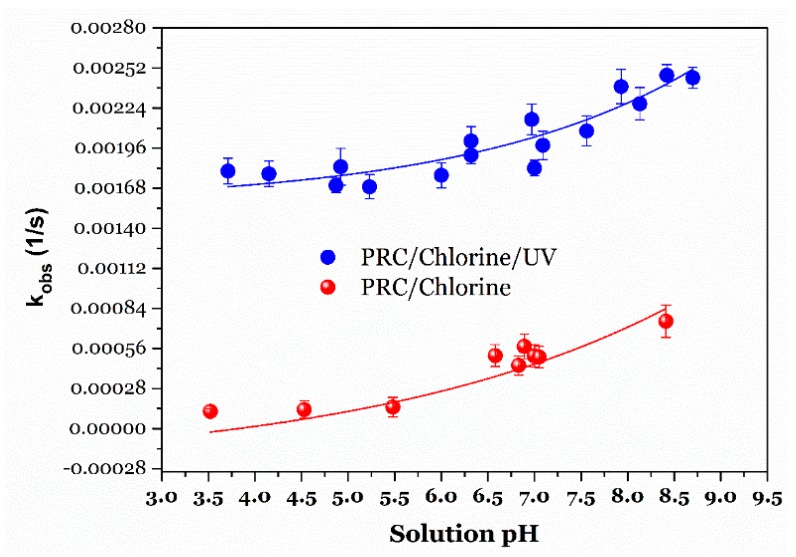
Effect of solution pH on PRC degradation rate constant by NaClO and NaClO/UV (Experimental conditions: UV photon flux = 3.41 × 10^−6^ Einstein/s, [PRC]_o_ = 10 µM, and [NaClO]_o_ ≈ 100 µM).

When the UV/chlorine process is operated to degrade the pollutant PRC, the controlled pH parameter plays an important role in distributing the existing forms of free chlorine Cl_2_, HOCl, and OCl^−^. To explore the impacts of solution pH values on the PRC degradation efficiency by the UV/chlorine process and chlorination alone, a series of experiments with the following conditions was carried out at different pH solutions from 3.5 to 8.5. As shown in Figure 5, the observed rate constants generally increased within increasing solution pH. Furthermore, the observed rate constants of the UV/chlorine-degraded PRC process were remarkably (approximately four times) higher than those of NaClO-degraded PRC one. The result confirmed that the PRC degradation occurred rapidly under the conditions of the combined UV and NaClO process.

Notably, when the solution pH was higher than 7.0, the *k*_obs_ values were remarkably increased by approximately 1.5 times for the two processes. This is because during the chlorination, the oxidant constituents and paracetamol species might be strongly dependent on their p*K*_a_ values. Paracetamol contains a phenol functional group in its structure, so its p*K*_a_ value is often around 9.5, suggesting that the existence of paracetamol in the ionized form seems negligible within the studied pH solutions (3.5–8.5). Moreover, the p*K*_a_ value of HOCl is approximately 7.5 at 25 °C. Therefore, when pH solutions were higher that its p*K*_a_, the dissociation of HOCl will occur to form the OCl^−^ ions (Equation (5)) [15,22]. The results suggested that OCl^−^ can contribute to degrading PRC faster than HOCl did. Zhu and coworkers [15] also found that ClO^−^ was a stronger oxidant than HOCl during the chlorination process of some secondary amides with the phenacetin structure. 

### 3.5. Effect of Water Matrices of Inorganic Ions and Natural Organic Matters

**Figure 6:**
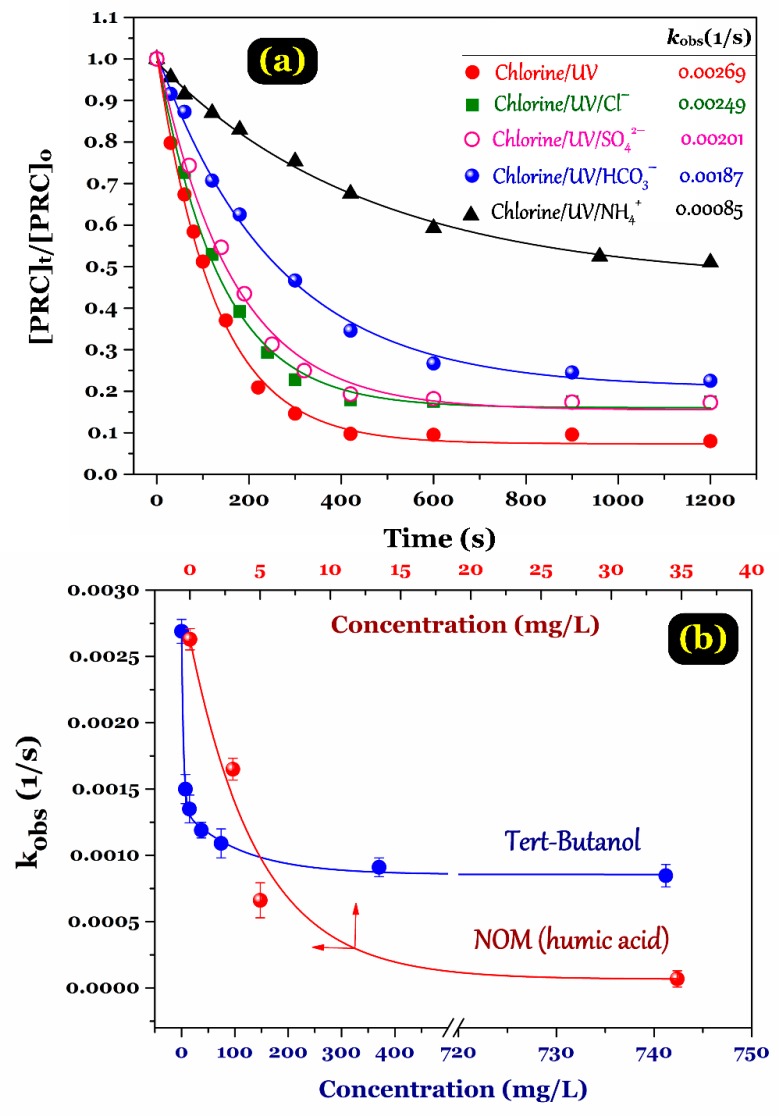
(**a**) Effect of some co-existing inorganic ions on the PRC degradation efficiency and rate (concentrations of each ion: 100 µM); and (**b**) effect of co-existing NOM (humic acid) and a ^•^OH scavenger (*tert*-butanol) on the PRC degradation rate constant (experimental conditions: [PRC]_o_ ≈ 10 µM, [NaClO]_o_ = 100 µM, pH = 7.0, and UV photon fluxes = 3.14 × 10^−6^ photon fluxes).

The most common inorganic ions existing in water bodies are ammonium (NH_4_^+^), nitrate (NO_3_^−^), chloride (Cl^−^), sulphate (SO_4_^2−^), and carbonate (CO_3_^2^^−^). These ions have an inevitable influence on the reaction mechanism and the formation of free radicals generated from the UV/chlorine system. These inorganic ions have strongly reactive radical (^•^OH, ^•^Cl, and ClO^•^) scavenging capacities, creating less reactive radicals, such as SO_4_^•^^−^ and CO_3_^•^^−^. Generally, the presence of Cl^−^, SO_4_^2^^−^, HCO_3_^−^, and NH_4_^+^ ions caused a decrease in the PRC degradation efficiency and rate constant (Figure 6a). The effects of foreign ions on the UV/chlorine process indicated the following order: no ion > chloride > sulphate > bicarbonate > ammonium presence. Among the selective anions, bicarbonate had the most negative impact on the PRC degradation efficiency and rate constant because the HCO_3_^−^ ions were a strong scavenger of ^•^OH radicals in the UV/chlorine system [22].

For water quality, natural organic matter (NOM) might provide a convenient source for the formation of disinfection by-products (DBPs) (as precursors) and microbial reproduction (as organic food source) in municipal water distribution systems. As reported in the literature, the presence of NOM in water matrices has a great influence on the degradation process of aromatic pollutants through: (1) free radical scavenging capacity (also known as radical scavenging effect) and (2) competitively interacting with the photons during the UV/chlorine process (UV filtering effect) [15,22,23]. For comparative purposes, a water matrix of humic acid was used as NOM source; meanwhile, *tert*–butanol (*tert*-B) is well known as a ^•^OH scavenger. The effects of different NOM concentrations on PRC degradation by the UV/chlorine process are plotted in Figure 6b. The results demonstrated that the PRC degradation process was inhibited when the NOM concentration was higher than 5 mg/L. Unlike the ^•^OH scavenger *tert*-B, NOM is a known radical scavenger that possibly reacts with both ^•^OH and ^•^Cl radicals [24,25]. Therefore, NOM indicated a greater inhibition effect of PRC degradation than *tert*-butanol. The influence of NOM present in the degradation process of organic compounds under the UV/chlorine process has been highlighted by other scholars [15,20,24,25]. 

**Figure 7:**
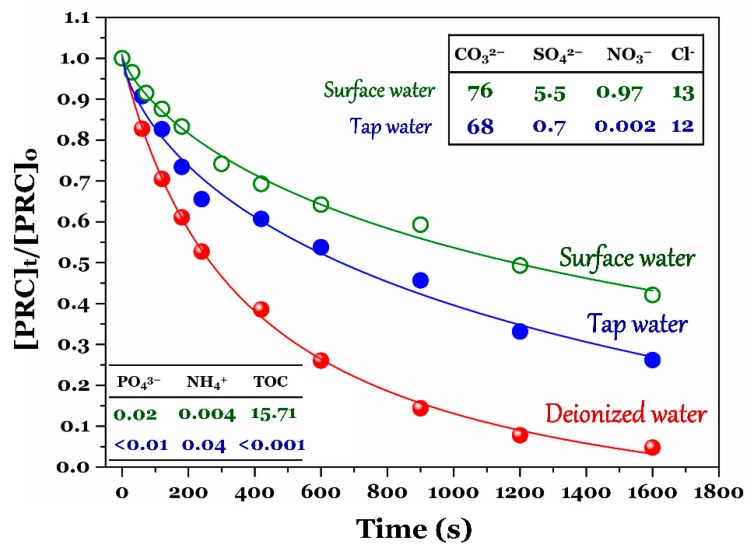
Comparison of PRC degradation efficiency under different kinds of water samples (experimental conditions: [PRC]_o_ ≈ 10 µM, [NaClO]_o_ = 100 µM, pH = 7.0, and UV photon flux = 3.14 × 10^−6^ photon fluxes).

Notably, the PRC degradation efficiency in different kinds of water samples was also examined. Some typical quality parameters of surface water and tap water are summarized in Figure 7. The results showed that the degradation efficiency of PRC within 10 min by the UV/chlorine process followed the order: approximately 74% (deionized water) > 46% (tap water) > 36% (surface water collected at Ho Tay lake, Ha Noi, Vietnam). A lower degradation efficiency of PRC in tap and surface water environments resulted from the higher concentrations of NH_4_^+^, Cl^−^, SO_4_^2−^, PO_4_^3−^, and CO_3_^2−^, especially DOM. The conclusion is well consistent with the result in Figure 6.

### 3.6. Optimization of PRC Degradation by Response Surface Methodology

#### 3.6.1. Modeling of Paracetamol Degradation

The result of removal efficiency of PRC (RE%) after 10 min with different parameters (UV, pH, NaOCl, and DOM variables) obtained through the RSM experimental design is shown in Table S1. The results were used for analysing the statistics and predicting the regression equation with the software MODDE 12 Pro. The regression coefficient values for coded variables of the polynomial functions are shown in Table S1. In addition, the statistical Student’s test was used to evaluate the significance of the regression coefficients (Table 3). Moreover, the quadratic regression equation of response functions for PRC removal efficiency was obtained after removing nonsignificant regression coefficients.

The quadratic regression equation (Equation (10)) of the response function for removal efficiency was obtained after removing nonsignificant regression coefficients. The coefficients (*X*_1_, *X*_2_, *X*_3_, and *X*_4_) have been defined in Table 1. The coefficient sign is helpful to evaluate a rapid analysis of the parametrical effects of the model variables on the responses. As shown in Equation (10), the negative coefficients (*X*_4_, *X*_2_^2^, and *X*_3_^2^) indicated an unfavorable effect on RE%; while, the positive coefficients (*X*_1_ and *X*_3_) referred to a favorable effect on RE%. The parametric coefficient with its value close to zero (*P*-value >0.05) indicated a lower relative intensity than the coefficients (*X*_1_, *X*_2_, *X*_3_, *X*_2_^2^, *X*_3_^2^, and *X*_4_):

RE% = 47.406 + 3.444 *X*_1_ + 14.398 *X*_3_ − 7.006 *X*_4_ - 9.983 *X*_2_^2^ − 5.420 *X*_3_^2^ (10)

**Figure 8:**
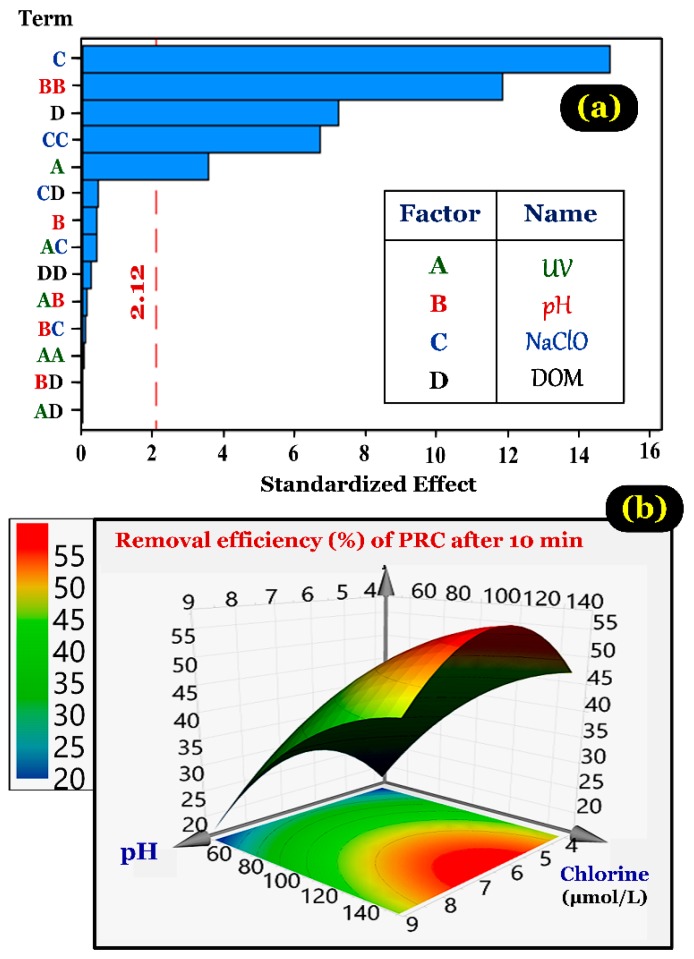
(**a**) Pareto chart of standardized effects (response is removal efficiency, α = 0.05); and (**b**) typical response surfaces and contour plots from central composite circumscribed design showing the interactive effect of pH and NaOCl on the removal efficiency of PRC in water.

However, the individual *P*-value is insufficient to evaluate the statistical significance of the predictors and develop the model. Therefore, the ANOVA with 95% confidence intervals for RE% was applied, and the results are represented in Table 4 and Figure 8a. Table 4 provides the analysis results of the variance (ANOVA) for the quadratic regression and linear component equations for the variables (i.e., UV, pH, NaOCl, and DOM). The statistical significance of the model was confirmed by the determination coefficient (*R*^2^), the adjusted determination coefficient (*R*^2^-adj), and the Fisher distribution (*F* test). 

The results demonstrated that the determination coefficient value for RE% was close to unity (*R*^2^ = 0.954), which is agreement with the *R*^2^-adj one (Table 4). The lack of fit was also calculated to measure how to the model fitted the data. Thus, the *P*-value of the lack of fit for RE% was 0.361. An insignificant lack of fit (*P* >0.10) is a desirable property because it suggests that the model fitted the data well. The calculated *F* value for the full quadratic regression equations of RE% was 1.317 [<3.874 for the *F*(0.95, 19, 6) value], indicating that the model fitted well with the experimental data. The results of ANOVA indicated that the quadratic regression equation models for the response of UV, pH, NaOCl, and DOM variables provided a good statistical validation for predicting experiments with a valid concentration region.

#### 3.6.2. Optimization of Paracetamol Degradation

The highest PRC removal efficiency after 10 min under the optimal experiment conditions was predicted by the RSM. The four condition predictors include UV light intensity (*X*_1_), pH value (*X*_2_), initial NaOCl concentration (*X*_3_), and DOM concentration (*X*_4_). Using the numerical optimization function of the statistical software MODDE 12.1, the results indicated that the highest PRC removal efficiency (77.87%) was obtained at the UV photon fluxes (13.6393 × 10^−6^ Einstein/s), pH (6.43), chlorine concentration (166.36 μmol/L), and DOM concentration (0.500124 mg/L). A typical example of multiple response approach is provided in Figure 8b obtained from the desirability 3D response surface for two typical variables (i.e., pH and chlorine) by maximizing the PRC removal efficiency at the optimal factors. 

Moreover, the attained optimal conditions were tested in another experimental run to validate the responses. The result demonstrated that 75.67% of PRC in solution was removed within 10 min. This confirmed the model reliability and accuracy because this removal efficiency lies 73.89% and 77.87% (the 95% confidence interval). Therefore, the modelling result can serve to estimate the removal efficiency of paracetamol with a high level of accuracy.

### 3.7. Degradation Pathways and Transformation Products Identification

Previous studies have shown that the active radicals (i.e., ^•^OH, ^•^Cl, and ClO^•^) are proved as indispensable factors in the degradation process of aromatic pollutants [15,20,22,23,25,26]. In this study, the degradation of PRC by the UV/NaOCl system was investigated by incorporating LC-MS/MS analysis and Compound Discoverer 2.0. The results demonstrated that the combination of UV and NaOCl can notably enhance the degradation of PRC and the formation of chloro compounds compared to either UV or NaOCl alone. Approximately 90% of PRC was degraded within 10 min, which is shown by comparing some signal peak areas over time on the LCMS chromatogram (Figure S1). 

The results indicated that **1** (PRC) was identified with a retention time (RT) of 6.0 min corresponding with the mass-to-charge ratio (*m*/*z*), with the precursors being 152.06367 [M + H] and 174.05312 [M + Na] and the fragment ion peak at *m*/*z* being 110.06059. As the reaction proceeded, the peak intensity of PRC decreased simultaneously with an increase in the intensity of the other peaks with retention times (from 2 to 11 min), indicating that numerous chloro compounds were formed within 10 min. The structures of these chloro PRC derivatives were determined by the unique and identifiable ratios of isotopic peaks found in chloro compounds and their MS/MS chromatograms (Table S2 and Figure S2). 

**Figure 9:**
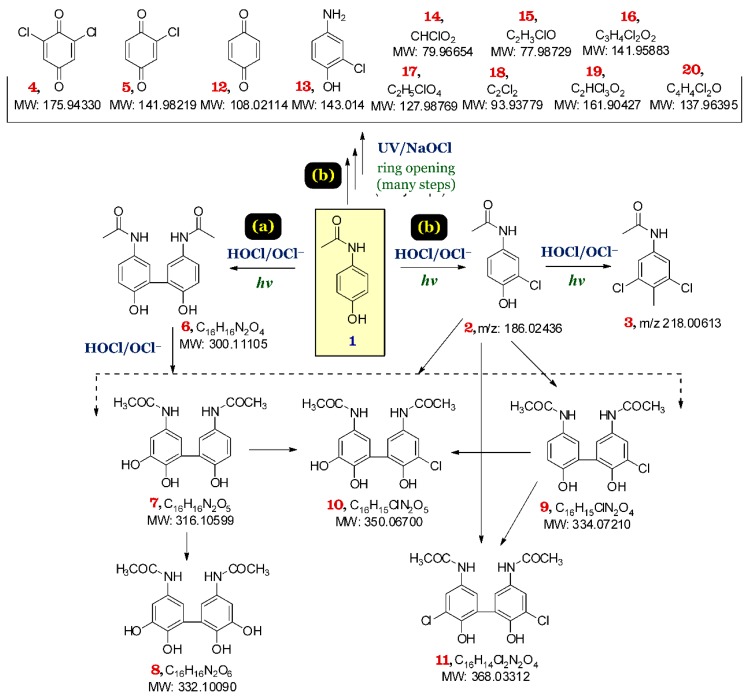
Possible pathways of PRC degradation under the UV/chlorine advanced oxidation process.

In addition, two expected compounds— monochloro-PRC **2** and dichloro-PRC **3**, with retention times of 7.5 min and 8.7 min, respectively—were detected through the corresponding mass-to-charge ratio (Figure 9). The *m*/*z* of the compound former was 186.02436 (100%) and 188.02436 (32%); meanwhile, that of the latter was 218.00613 (100%), 220.00613 (64%), and 222. 00623 (10%). The results are well consistent with the conclusion of Cao and colleagues [34] for the mass spectrometer chromatogram of the monochloro-PRC and dichloro-PRC derivatives.

On the basis of the structures of these aforementioned compounds, the mechanisms of degradation could be explained by the attack of ^•^OH or ^•^Cl radicals on the aromatic ring of PRC molecules, leading to the formations of *ortho*-, *meta*-, or *para*-chlorinated PRC derivatives. An analogous result was reported by Vogna and co-workers [35]. Moreover, the (186.02436 *m*/*z*) fragment was detected at 144.01430 *m*/*z* (losing a CH_3_CO group) and 109.02114 *m*/*z* (losing CH_3_CO and Cl groups). Meanwhile, the (219.00613 *m*/*z*) fragment was detected at 175.98773 *m*/*z* (losing CH_3_CO group) and 143.01 *m*/*z* (losing CH_3_CO and Cl groups). 

Along with the decreasing peak intensity of PRC in the reaction mixture, an increase in the peak intensities of compounds **2** and **3** occurred concurrently. Notably, the peaks of the two compounds **2** and **3** were not detected in the mass spectrum after 10 min (Figure S1). The possible reasons were due to the transformation of **2** into **3**; and the transformation of **2** into **7** (317.10599 *m*/*z*), **8** (333.10090), **9** (335.07210), **10** (351.06700), and then to **11** (369.03312). 

Notably, several organic compounds with molecular weights smaller than PRC were identified along with the existing molecular weights. The presence of the small molecular weight compounds resulted from the breakdown of PRC during the degradation process. These compounds were chloro-derivatives—138.96395 *m*/*z* (two chlorine atoms), 128.98769 *m*/*z* (one chlorine atom), 80.96654 *m*/*z* (chlorine atom), and 78.98729 *m*/*z* (chlorine atom)—that were detected at retention times ranging from 2.3 to 2.7 min in the LC-MS positive mode. In contrast, the LC-MS negative mode indicated the identical organic compounds, such as 174.94330 *m*/*z* (two chlorine atoms), 140.98219 *m*/*z* (one chlorine atom), and 107.02114 *m*/*z* (no chlorine atom) at retention times of 5.5 to 6.0 min as well as 160.90427 *m*/*z* (three chlorine atoms), 140.95883 *m*/*z* (two chlorine atoms), and 92.93779 *m*/*z* (two chlorine atoms) at retention times of 2.0 to 2.6 min (Figure 9).

In summary, the two probable degradation pathways of PRC under the UV/chlorine system conditions, which were identified by the LC-MS technique, are summarised in Figure 9. They include the breakdown of PRC followed by chlorination (pathway **a**) and the chlorination followed the breakdown of the resulting chlorinated compounds (pathway **b**). Notably, some organic compounds could be also detected in trace levels in pathway **b** (i.e., compounds **4**, **5**, **19**, **20**, **21**, and **22**; Figure S2). 

## 4. Conclusions

We have studied the degradation of PRC by the UV/chlorine in water media under different operational conditions and elucidated the main intermediates. It can be experimentally concluded that:

- The PRC degradation rate constant (*k*_obs_) follows pseudo-first-order kinetics. Under the same experimental conditions, the *k*_obs_ values obtained by the UV/chlorine process were overwhelmingly higher than those by UV/H_2_O_2_, chlorination alone, and UV alone.
- The operational parameters most positively effecting the degradation rate constant of PRC were NaClO dosage, followed by UV irradiation and solution pH value. In contrast, the presence of inorganic ions and natural organic matters significantly inhibited the PRC degradation process.
- The reactive radicals ^•^OH, ^•^Cl, and ClO^•^ play an important role in degrading PRC by the UV/chlorine advanced oxidation process.
- Response surface methodology was applied to evaluate the interaction of four independent variables (chlorine, UV, pH, and DOM). The result indicated that the highest PRC removal was obtained under the optimal conditions of UV photon flux (13.6 × 10^−6^ Einstein/s), pH (6.43), chlorine concentration (166 μmol/L), and DOM concentration (0.50 mg/L).
- Twenty compounds and structures have been proposed using LC-MS/MS in combination with the software Compound Discoverer 2.0. The *ortho*-position proved to be the major position of radical substitution. Monochloro-PRC and dichloro-PRC were identified as the two major derivatives, which concentration increased at the beginning and then subsequently decreased with the increase of the other PRC derivatives.
- We have presumed the chlorination pathway to be the primary mechanism of the PRC degradation generating the major compounds. A secondary breakdown pathway led to the formation of several unsaturated and carboxylic compounds that were detected using LC-MS.
- The toxicity of PRC transformation products and its variation during the UV/chlorine process should be further investigated and evaluated.

## Figures and Tables

**Table 1:** Parameters of four variables.

Symbol	Variable	Unit	Coded Variable and Independent Variable				
			−α	−1	0	1	+α
***X*_1_**	*I*_o_	Einstein/s	0	3.41	6.82	10.2	13.6
***X*_2_**	pH	—	1.5	4.0	6.5	9.0	11.5
***X*_3_**	[NaOCl]	μM	0	50	100	150	200
***X*_4_**	DOM	mg/L	0.5	2.0	3.5	5.0	6.5

Note: UV light intensity (*I*_o_), chlorine concentration ([NaOCl]), and dissolved organic matter (DOM).

**Table 2:** Contribution percentage of UV, chlorine, and reactive radicals to the PRC degradation by the UV/chlorine process under different UV light intensities (*I*_o_).

*I*_o_ (10^−6^ E/s)	The Observed Degradation Rate Constant (10^−3^/s)			Contribution (%)		
	PRC/UV	PRC/Chlorine	PRC/UV/Chlorine	UV	Chlorine	Radicals
3.41	0.195	0.121	2.01	9.70	6.02	84.3
6.82	0.381	0.121	3.36	11.34	3.60	85.1
10.23	0.571	0.121	4.81	11.87	2.52	85.6

Note: Experimental conditions: [PRC]_o_ = 10 μmol/L, [NaClO] = 100 μmol/L, pH = 7.0, and *T* = 25 °C.

**Table 3:** Regression coefficients values (coded variables) of the polynomial model of responses for removal efficiency of PRC after 10 min.

Removal Efficiency	Coeff. SC	Std. Err.	T-Value	*P*	Conf. Int (±)
**Constant**	46.41	2.009	23.14	1.02 × 10^−13^	4.258
**UV light intensity**	3.444	1.085	3.18	0.00589	2.299
**pH**	−0.423	1.085	−0.39	0.702	2.299
**Chlorine**	14.39	1.085	13.29	4.72 × 10^−10^	2.299
**DOM**	−7.006	1.085	−6.46	7.91 × 10^−6^	2.299
*I*_o_*I_o_	0.671	0.994	0.68	0.509	2.107
pH*pH	−9.879	0.994	−9.95	2.98 × 10^−8^	2.107
NaClO*NaClO	−5.317	0.994	−5.36	6.51 × 10^−5^	2.107
DOM*DOM	0.403	0.994	0.41	0.690	2.107
*I*_o_*pH	−0.184	1.329	−0.14	0.892	2.817
*I*_o_*NaClO	−0.516	1.329	−0.39	0.703	2.817
*I*_o_*DOM	0.009	1.329	0.00	0.995	2.817
pH*NaClO	−0.141	1.329	−0.11	0.917	2.817
pH*DOM	0.034	1.329	0.02	0.979	2.817
NaClO*DOM	0.541	1.329	0.41	0.689	2.817
**Model Summary**					
**N = 31**	Q^2^ =	0.790		Cond. No. =	4.686
**DF = 16**	R^2^ =	0.957		RSD =	5.315
	R^2^-adj. =	0.919		Confidence =	0.95

Note: DF = degree of freedom; Adj SS = adjusted sum of square; Adj MS = adjusted mean square; SD = standard deviation. UV light intensity (*I*_o_), chlorine concentration ([NaOCl]), and dissolved organic matter (DOM).

**Table 4:** Analysis of variance (ANOVA) for variables and the regression function.

Removal Efficiency (10 min)	DF	SS	MS (Variance)	*F*	*P*	SD
**Total**	31	49,462.5	1595.56			
**Constant**	1	39,026.2	39026.2			
**Total corrected**	30	10,436.3	347.878			18.6515
**Regression**	6	9958.56	1659.76	83.3734	0.000	40.7401
**Residual**	24	477.781	19.9075			4.46179
**Lack of fit**	19	385.373	20.2827	1.3169	0.361	4.62705
**Pure error**	6	92.4086	15.4014			3.92447
	N = 31	Q^2^ =	0.908	Cond. no. =	2.955	
	DF = 24	R^2^ =	0.954	RSD =	4.462	
		R^2^-adj. =	0.943			

## Supplementary Materials

The following are available online at http://www.mdpi.com/1660-4601/15/12/2637/s1, Table S1: The RSM experiment design matrix and experimental results; Table S2: Accurate mass measurement of product ions of PRC and its transformed products identified by LC/MS/MS; Figure S1: Change of signal peak areas of main transformation products within 10 min; and Figure S2: Result of liquid chromatography-tandem mass spectrometry (LC-MS/MS) of proposed compound.
