# Supplementary material for: Degradation of Paracetamol by an UV/Chlorine Advanced Oxidation Process: Influencing Factors, Factorial Design, and Intermediates Identification
Source: Int J Environ Res Public Health. 2018 Nov 25;15(12):2637. doi: 10.3390/ijerph15122637 (PMC6313806; doi:10.3390/ijerph15122637)
Supplement: Supplementary file 1 [file ijerph-15-02637-s001.pdf]

Supporting information:

Degradation of Paracetamol by an UV/Chlorine Advanced Oxidation Process: Influencing Factors, Factorial Design, and Intermediates Identification

Yen Hai Dao[1], Hai Nguyen Tran[2*], Thien Thanh Tran-Lam[1], Trung Quoc Pham[1], Giang Truong Le[1]**

[1]Institute of Chemistry, Vietnam Academy of Science and Technology, 18 Hoang Quoc Viet, Cau Giay, Ha Noi 100000, Vietnam

[2]Institute of Research and Development, Duy Tan University, Da Nang 550000, Vietnam

----

*Correspondence: trannguyenhai@duytan.edu.vn; trannguyenhai2512@gmail.com; Tel.: +84-0967-070-062

**Correspondence: hoasinhmoitruong.vast@gmail.com; Tel.: +84-98-585-9795

Table S1. The RSM experiment design matrix and experimental results

Exp	Run order	Coded variable				Independent variables				Removal efficiency (%)	
		X_1	X_2	X_3	X_4	UV	pH	NaOCl	DOM	Experiment	Model
1	1	-1	-1	-1	-1	3.41	4	50	2	19.4	21.2
2	18	1	-1	-1	-1	10.23	4	50	2	25.7	28.1
3	22	-1	1	-1	-1	3.41	9	50	2	18.9	21.2
4	19	1	1	-1	-1	10.23	9	50	2	24.6	28.1
5	6	-1	-1	1	-1	3.41	4	150	2	50.8	50.0
6	9	1	-1	1	-1	10.23	4	150	2	55.9	56.9
7	7	-1	1	1	-1	3.41	9	150	2	49.6	50.0
8	14	1	1	1	-1	10.23	9	150	2	53.6	56.9
9	4	-1	-1	-1	1	3.41	4	50	5	8.6	7.2
10	17	1	-1	-1	1	10.23	4	50	5	15.5	14.0
11	12	-1	1	-1	1	3.41	9	50	5	7.8	7.2
12	20	1	1	-1	1	10.23	9	50	5	14.2	14.0
13	13	-1	-1	1	1	3.41	4	150	5	42.5	36.0
14	28	1	-1	1	1	10.23	4	150	5	46.8	42.8
15	8	-1	1	1	1	3.41	9	150	5	41.6	36.0
16	16	1	1	1	1	10.23	9	150	5	45.2	42.8
17	26	-2	0	0	0	0.00	6.5	100	3.5	38.5	40.5
18	11	2	0	0	0	13.64	6.5	100	3.5	58.7	54.3
19	5	0	-2	0	0	6.82	1.5	100	3.5	6.5	7.5
20	29	0	2	0	0	6.82	11.5	100	3.5	6.3	7.5
21	21	0	0	-2	0	6.82	6.5	0	3.5	1.1	18.6
22	23	0	0	2	0	6.82	6.5	200	3.5	48.2	76.2
23	3	0	0	0	-2	6.82	6.5	100	0.5	70.5	39.7
24	15	0	0	0	2	6.82	6.5	100	6.5	24.6	11.7
25	2	0	0	0	0	6.82	6.5	100	3.5	50.2	47.4
26	10	0	0	0	0	6.82	6.5	100	3.5	45.5	47.4
27	27	0	0	0	0	6.82	6.5	100	3.5	46.7	47.4
28	31	0	0	0	0	6.82	6.5	100	3.5	42.5	47.4
29	24	0	0	0	0	6.82	6.5	100	3.5	41.2	47.4
30	30	0	0	0	0	6.82	6.5	100	3.5	52.3	47.4
31	25	0	0	0	0	6.82	6.5	100	3.5	46.5	47.4

Table S2. Accurate mass measurement of product ions of PRC and its transformed products identified by LC/MS/MS

Compound symbol	m/z, [M+/-X]	RT	Proposed formula
**1**	**152.06334, [M+1]**	**6.10**	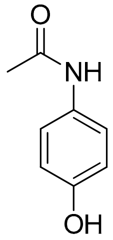<br>Chemical Formula: $C_8H_9NO_2$<br>Exact Mass: 151.06334
**2**	**186.02430, [M+1]**	**7.56**	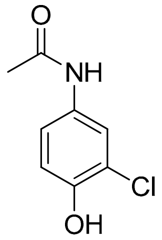<br>Chemical Formula: $C_8H_8ClNO_2$<br>Exact Mass: 185.02430
**3**	**219.00610, [M+1]**	**8.76**	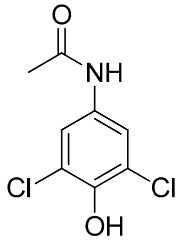<br>Chemical Formula: $C_8H_7Cl_2NO_2$<br>Exact Mass: 218.00610
**4**	**174.94330, [M-1]**	**5.59**	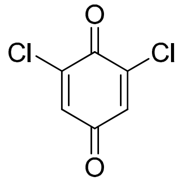<br>Chemical Formula: $C_6H_2Cl_2O_2$<br>Exact Mass: 175.94330
**5**	**301.11105, [M+1]**	**7.01**	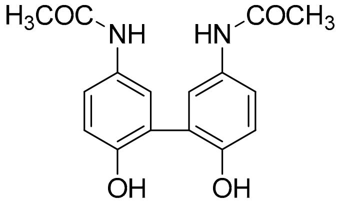<br>Chemical Formula: $C_{16}H_{16}N_2O_4$<br>Exact Mass: 300.11105

**6**	**335.07210, [M+1]**	**7.98**	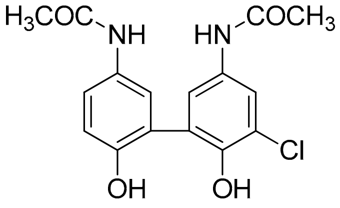<br>Chemical Formula: $C_{16}H_{15}ClN_2O_4$<br>Exact Mass: 334.07210
**7**	**317.10599, [M+1]**	**8.65**	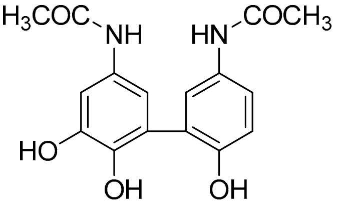<br>Chemical Formula: $C_{16}H_{16}N_2O_5$<br>Exact Mass: 316.10599
**8**	**369.03312, [M+1]**	**8.66**	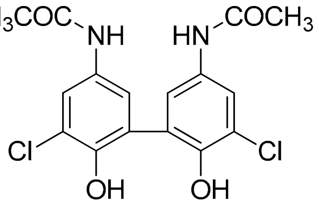<br>Chemical Formula: $C_{16}H_{14}Cl_2N_2O_4$<br>Exact Mass: 368.03312
**9**	**351.06700, [M+1]**	**14.72**	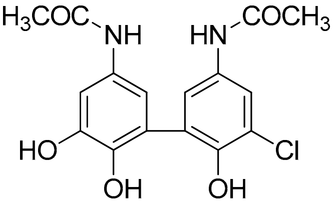<br>Chemical Formula: $C_{16}H_{15}ClN_2O_5$<br>Exact Mass: 350.06700
**10**	**144.01400, [M+1]**	**8.67**	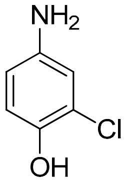<br>Chemical Formula: C_6H_6ClNO<br>Exact Mass: 143.01400
**11**	**140.98219, [M-1]**	**9.11**	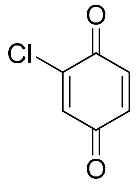<br>Chemical Formula: $C_6H_3ClO_2$<br>Exact Mass: 141.98219

**12**	**107.02114, [M-1]**	**6.00**	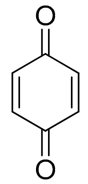 Chemical Formula: $C_6H_4O_2$ Exact Mass: 108.02114
**13**	**144.01400, [M+1]**	**10.1 and 10.5**	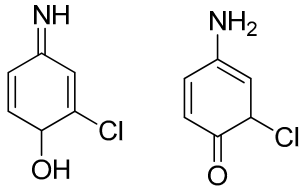 Chemical Formula: C_6H_6ClNO Exact Mass: 143.01400
**14**	**160.90427; [M-1]**	**2.6**	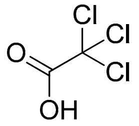 Chemical Formula: $C_2HCl_3O_2$ Exact Mass: 161.90427
**15**	**140,95883; [M-1]**	**2,52**	Chemical Formula: $C_3H_4Cl_2O_2$ Exact Mass: 141.95883
**16**	**92,93779; [M-1]**	**2,50**	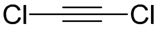 Chemical Formula: C_2Cl_2 Exact Mass: 93.93779
**17**	**138,96395; [M+1]**	**2,42**	Chemical Formula: $C_4H_4Cl_2O$ Exact Mass: 137.96395
**18**	**128,98769; [M+1]**	**2,60**	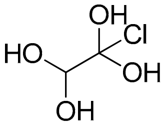 Chemical Formula: $C_2H_5ClO_4$ Exact Mass: 127.98769
**19**	**80,96654; [M+1]**	**2,50**	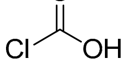 Chemical Formula: $CHClO_2$ Exact Mass: 79.96654
**20**	**78,98729; [M+1]**	**2,32**	Chemical Formula: C_2H_3ClO Exact Mass: 77.98729

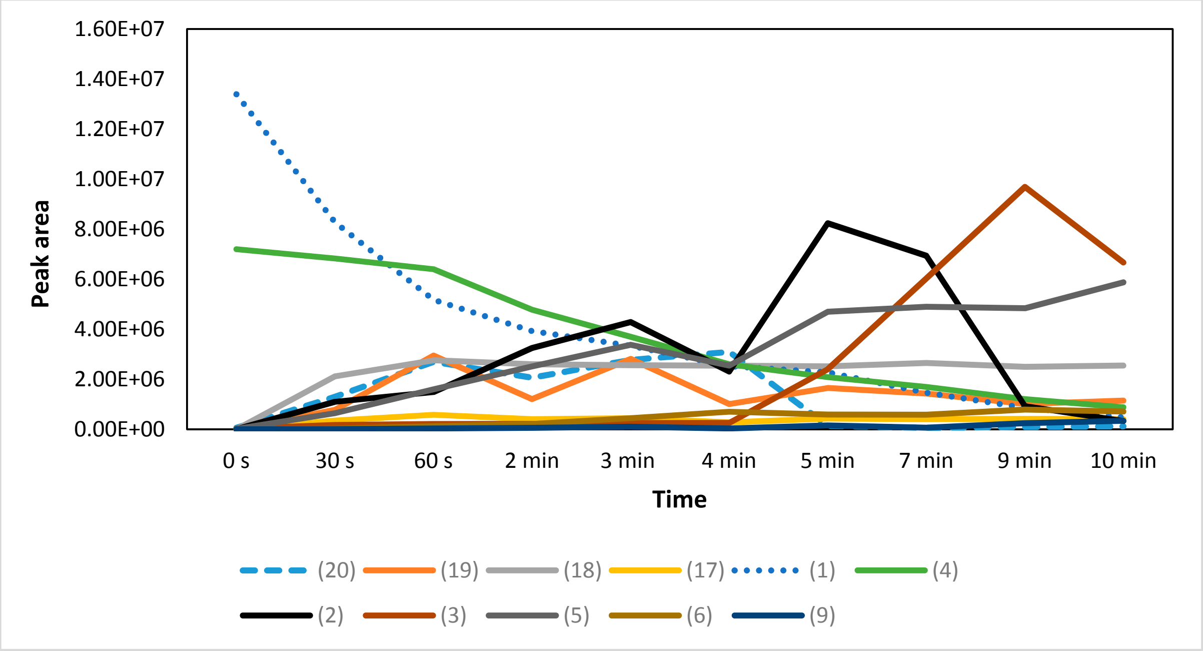

Figure S1. Change of signal peak areas of main transformation products within 10 min

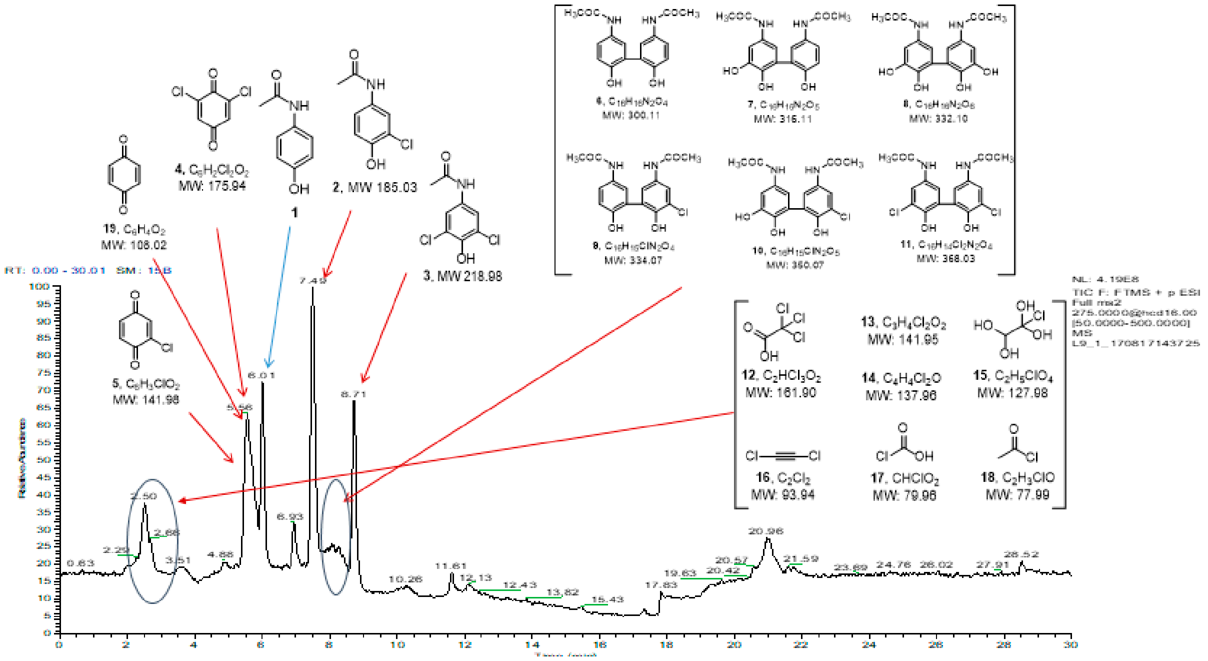

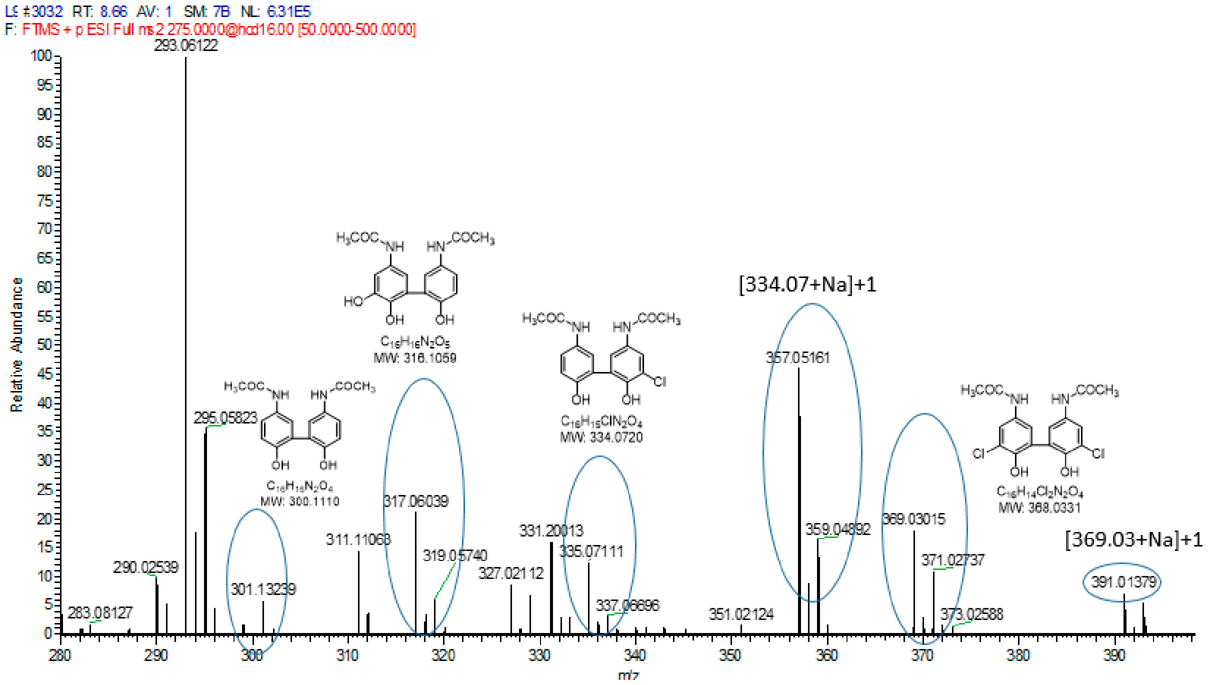

Section S2. Result of liquid chromatography-tandem mass spectrometry (LC-MS/MS) of proposed compound
